# Supplementary material for: Modeling cannabinoids from a large-scale sample of Cannabis sativa chemotypes
Source: PLoS One. 2020 Sep 1;15(9):e0236878. doi: 10.1371/journal.pone.0236878 (PMC7462266; doi:10.1371/journal.pone.0236878)
Supplement: S1 File — (DOCX) [file pone.0236878.s001.docx]

**Supplementary Information**

**Modeling cannabinoids from a large-scale sample of *Cannabis sativa* chemotypes**

Daniela Vergara^1*^, Reggie Gaudino^2^, Thomas Blank^2^, and Brian Keegan^3*^

**Methods**

**Missing data and imputation**

**S1 Fig.** **Distribution of cases based on number of missing cannabinoid observations**. Only 153 cultivars contain all cannabinoid information, while 1,386 samples miss the information of one of the cannabinoids, 3,977 of two cannabinoids and so on for a total of 17,600 data points analyzed.

The density-based spatial clustering of applications with noise (DBSCAN) algorithm (1) was used to classify points to four clusters using a Euclidian distance metric, a minimum of 75 samples per cluster, and an EPS step size of 0.5.

**Geographic space analysis**

Due to the amount of missing data for CBN, THCV, and CBDV, we did not include these three cannabinoids in the geographic space analysis. We also excluded CBDV and THCB due to the poor understanding of their biochemistry, and CBN because it’s a breakdown product (2-4). Since only 2014 had samples for three states, we analyzed the cannabinoids of these locations only for that year. We had 1,364 samples from California, 936 from Colorado, and 244 from Washington.

We performed a one-way ANOVA for each of four cannabinoids – CBG, CBC, THC, and CBD- as the response variable and state as a factor, with a posterior posthoc analysis to determine cannabinoid level differences by year for each state.

**Analysis through time**

California was the only state that had 16,41 samples from every year for eight years 2011-2018 divided as follows: 361 from 2011; 574 from 2012; 827 from 2013; 1,364 from 2014; 1,113 from 2015; 5 from 2016; 5,727 from 2017; and 6,447 from 2018. Therefore, our analysis of cannabinoid change through time was only performed with samples from this state.

With a one-way ANOVA with each of four cannabinoids – CBG, THC, CBD, and CBC- as the response variable and year as a factor, and with a posterior posthoc analysis we determined whether cannabinoids differ in California in these 8 years.

**Results**

**Geographic space analysis**

Despite the disparity in sample size, we find overall differences in cannabinoids for the three states, California, Colorado, and Washington, for the 2014 subset (figure S2). CBG (figure S2A) shows significant overall variation (ANOVA p<0.0001, F=13.09), and the posthoc assessment shows that the three states differ from each other with p-values of less than 0.03. Overall, THC (figure S2B) differs across the three states (ANOVA p<0.01, F = 33.74), and the posthoc analysis reveals that the three states are significantly different from each other. CBD (figure S2C) also differs overall (ANOVA p<0.0001, F = 4.837), however, the pairwise comparisons show significant differences only between California and Colorado. Finally, CBC (figure S2D) varies significantly across the three states (ANOVA p< 0.0001, F=45.08) but the posthoc test shows that California differs from Washington and Colorado, while Washington and Colorado are not significantly different from each other. Essentially, none of the three states have more or less of a particular cannabinoid.

 **S2 Fig**. **Distribution of four cannabinoids, CBG (A), THC (B), CBD (C), and CBC (D) across three states for 2014.** Pairwise comparisons for each panel: **A** California-Colorado p<0.03; California-Washington p<0.0001; Washington-Colorado p<0.01. **B** California-Colorado p<0.01; California-Washington p<0.0001; Washington-Colorado p<0.0001. **C** California-Colorado p<0.01; California-Washington p=NS; Washington-Colorado p=NS. **D** California-Colorado p<0.0001; California-Washington p<0.0001; Washington-Colorado p=NS.

**Analysis through time**

Since California was the only state that had samples from every year for eight years (Table S1), we only included this state for our time analysis for only four of the cannabinoids: CBG, THC, CBD and CBC. With our one way ANOVA with each of the cannabinoids as the response variable and year as a factor, and with a posterior posthoc analysis we found that overall, every single cannabinoid showed significant differences: CBG (F= 234.4, p <0.0001, figure S3A); THC (F = 158.9, p <0.0001, figure S3B); CBD (F= 72.66, p <0.0001, figure S3C); and CBC (F= 5.08, p < 0.03, figure S3D). However, there is not one year where all cannabinoids differ, or there is not one year that differs from all of the other years (Tables S2 and S3). For CBG (Table S2 upper half) or CBD (Table S3 upper half) no year is significantly different from the rest. However, 2015 for THC (Table S2 bottom half) shows significant differences from the rest of the years due to the low levels of the cannabinoid (figure S3B). Similarly, in 2018, CBC (Table S3 bottom half) differs significantly form the other years but due to its high levels (figure S3D). In general, we see no trend for time in increase or decrease of particular cannabinoids in California.

**S3 Fig. Distribution of four cannabinoids across eight years for the state of California.** CBG **(A)** has no year that differs significantly from all other years. 2015 was significantly lower in THC **(B)** production compared to the rest of the years. For CBD **(C),** no year was significantly different, and for CBC **(D)** 2018 had significantly higher levels when compared to the other seven years. All pair comparisons are given in tables S1 and S2.

**Table S1.** **Pairwise comparison between years for CBG (upper triangle) and THC (lower triangle).**

|  |  | CBG California | |  |  |  |  |  |  |
| --- | --- | --- | --- | --- | --- | --- | --- | --- | --- |
|  |  | 2011 | 2012 | 2013 | 2014 | 2015 | 2016 | 2017 | 2018 |
| THC California | 2011 | - | NS | <0.0001 | NS | NS | NS | <0.0001 | NS |
|  | 2012 | NS | - | <0.0001 | <0.05 | <0.001 | NS | <0.0001 | <0.001 |
|  | 2013 | NS | NS | - | <0.0001 | <0.0001 | NS | <0.0001 | <0.0001 |
|  | 2014 | NS | NS | NS | - | NS | NS | <0.0001 | NS |
|  | 2015 | <0.0001 | <0.0001 | <0.0001 | <0.0001 | - | NS | <0.0001 | NS |
|  | 2016 | NS | NS | NS | NS | <0.0001 | - | NS | NS |
|  | 2017 | NS | NS | NS | NS | <0.0001 | NS | - | <0.0001 |
|  | 2018 | NS | NS | <0.01 | NS | <0.0001 | NS | <0.0001 | - |

For both cannabinoids, there is no particular trend in any of the years (Figure S2).

**Table S2.** **Pairwise comparison between years for CBD (upper triangle) and CBC (lower triangle).**

|  |  | CBD California | |  |  |  |  |  |  |
| --- | --- | --- | --- | --- | --- | --- | --- | --- | --- |
|  |  | 2011 | 2012 | 2013 | 2014 | 2015 | 2016 | 2017 | 2018 |
| CBC California | 2011 | - | NS | <0.0001 | NS | NS | NS | <0.0001 | <0.0001 |
|  | 2012 | NS | - | <0.0001 | <0.03 | NS | NS | <0.0001 | <0.0001 |
|  | 2013 | <0.05 | NS | - | <0.003 | <0.001 | NS | NS | NS |
|  | 2014 | <0.03 | <0.0001 | <0.0001 | - | NS | NS | <0.0001 | <0.001 |
|  | 2015 | <0.0001 | <0.0001 | <0.0001 | NS | - | NS | <0.0001 | <0.01 |
|  | 2016 | NS | NS | NS | NS | NS | - | NS | NS |
|  | 2017 | NS | <0.001 | <0.0001 | NS | <0.003 | NS | - | <0.01 |
|  | 2018 | <0.0001 | <0.0001 | <0.0001 | <0.0001 | <0.0001 | NS | <0.0001 | - |

For both cannabinoids, there is no particular trend in any of the years (Figure S2).

**Dimensionality reduction**

Figure S4 is a projection of each of the four imputation methods using the t-SNE technique. The cluster labels using only the CBD and THC relationships in Figure 3 again capture the same clusters in these projections. The relative proximity of the four clusters in these projections appears to be dominated more by similarity in CBD than in THC: the high-CBD, high-THC (blue) is close to the high-CBD, low-THC (red) cluster while the low-CBD, high-THC (orange) and low-CBD, low-THC (purple) clusters are proximate. The iterative method (upper left) projected by t-SNE locates imputed values both in the high-THC, low-CBD (orange) cluster as well as a new cluster closer to the high-THC, high-CBD (blue) cluster. The multiple imputation method (upper right) creates two new distinctive clusters beyond the high-THC, low-CBD (orange) cluster. The k-nearest neighbors (lower left) again locates most of the imputed values inside the high-THC, low-CBD (orange) cluster. Finally, the values imputed from the soft imputation method (lower right) again have minimal overlap with any of the other clusters and form an entirely new cluster between the high-THC and low-CBD (orange) cluster and the high-THC and high-CBD (blue) cluster.

**S4 Fig.** **Projection of each of the four imputation methods using the t-SNE**

Even though Principal Component Analysis is the most common visualization technique in biology (5), PCA dimensional reduction leads to little or no differentiation between possible groups in these two dimensional mappings (Figure S5).
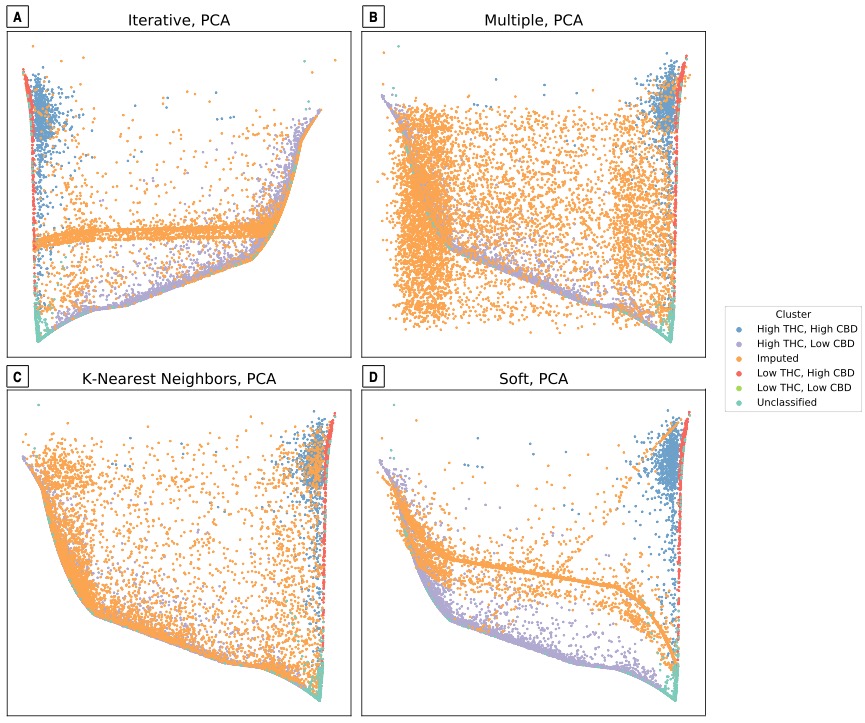
 **S5 Fig.** **Projection of each of the four imputation methods using PCA**

**References**

1. Ester M, Kriegel H-P, Sander J, Xu X, editors. A density-based algorithm for discovering clusters in large spatial databases with noise. Kdd; 1996.

2. Ross SA, ElSohly MA. CBN and∆ 9-THC concentration ratio as an indicator of the age of stored marijuana samples. Bulletin on Narcotics. 1997;49(50):139-.

3. Trofin IG, Dabija G, Vaireanu DI, Filipescu L. The influence of long-term storage conditions on the stability of cannabinoids derived from cannabis resin. Rev Chim Bucharest. 2012;63(4):422-7.

4. Turner CE, Elsohly MA. Constituents of cannabis sativa L. XVI. A possible decomposition pathway of Δ9‐tetrahydrocannabinol to cannabinol. Journal of heterocyclic chemistry. 1979;16(8):1667-8.

5. Ringnér M. What is principal component analysis? Nature biotechnology. 2008;26(3):303.
