# Supplementary material for: Modeling cannabinoids from a large-scale sample of Cannabis sativa chemotypes
Source: PLoS One. 2020 Sep 1;15(9):e0236878. doi: 10.1371/journal.pone.0236878 (PMC7462266; doi:10.1371/journal.pone.0236878)
Supplement: Attachment — Submitted filename: Response_to_reviewers.docx [file pone.0236878.s002.docx]

Reviewer #1: This is a very well written and interesting paper. I only have some very minor suggestions.
- The introduction is long. State clearly at the end the purpose of the study and the expectations.

**Thank you for your comment. We reduced the introduction and removed 500 words. We also state our expectations in the last paragraph of the introduction (lines 198-199)**

- The discussion should be clearly structured. Start with the new findings and not with the repeat of the methods.

**We deleted the sentences related to the methods and started the discussion with the findings from our study.**

What are the limitations of the study? Future studies? Conclusion paragraph.

**We appreciate your suggestion. We added a conclusion paragraph including limitations and moved a paragraph that included future directions into this new section.**

- "Relationship" is not a statistical term. Please use correlation or association.

**We changed the word relationship to correlation and association**

Reviewer #2: This work cluster cultivars and chemotypes together and show that cultivars cluster based on their chemotype. This work open a window for characterizing complex interactions in cannabinoid biosynthesis. Even the authors used a big number of cultivars and good bioinformatics tools, the work is very basic and not bring novel data or ideas.
I think this is a good solid work which can fit to Plose One policy of publication as the data is good and statistical analysis been performed appropriately.

3. Thank you for stating the following in the Financial Disclosure section:

'This research was supported by donations to the Agricultural Genomics Foundation, to the University of Colorado Foundation gift fund 13401977-Fin8 to Professor Nolan C. Kane, and is part of the joint research agreement between the University of Colorado Boulder and Steep Hill Inc.'

We note that one or more of the authors are employed by a commercial company:Steep Hill Inc.

1. Please provide an amended Funding Statement declaring this commercial affiliation, as well as a statement regarding the Role of Funders in your study. If the funding organization did not play a role in the study design, data collection and analysis, decision to publish, or preparation of the manuscript and only provided financial support in the form of authors' salaries and/or research materials, please review your statements relating to the author contributions, and ensure you have specifically and accurately indicated the role(s) that these authors had in your study. You can update author roles in the Author Contributions section of the online submission form.

Please also include the following statement within your amended Funding Statement.

“The funder provided support in the form of salaries for authors [insert relevant initials], but did not have any additional role in the study design, data collection and analysis, decision to publish, or preparation of the manuscript. The specific roles of these authors are articulated in the ‘author contributions’ section.”

If your commercial affiliation did play a role in your study, please state and explain this role within your updated Funding Statement.

2. Please also provide an updated Competing Interests Statement declaring this commercial affiliation along with any other relevant declarations relating to employment, consultancy, patents, products in development, or marketed products, etc.

Within your Competing Interests Statement, please confirm that this commercial affiliation does not alter your adherence to all PLOS ONE policies on sharing data and materials by including the following statement: "This does not alter our adherence to  PLOS ONE policies on sharing data and materials.” (as detailed online in our guide for authors [http://journals.plos.org/plosone/s/competing-interests](about:blank)) . If this adherence statement is not accurate and  there are restrictions on sharing of data and/or materials, please state these. Please note that we cannot proceed with consideration of your article until this information has been declared.

Please include both an updated Funding Statement and Competing Interests Statement in your cover letter. We will change the online submission form on your behalf.

Please know it is PLOS ONE policy for corresponding authors to declare, on behalf of all authors, all potential competing interests for the purposes of transparency. PLOS defines a competing interest as anything that interferes with, or could reasonably be perceived as interfering with, the full and objective presentation, peer review, editorial decision-making, or publication of research or non-research articles submitted to one of the journals. Competing interests can be financial or non-financial, professional, or personal. Competing interests can arise in relationship to an organization or another person. Please follow this link to our website for more details on competing interests: [http://journals.plos.org/plosone/s/competing-interests](about:blank)

**We added all suggested statements to our competing interests and funding sections. We want to clarify that the analyzed data for this project was provided by the company Steep Hill Inc. The company specializes in chemotype testing and we analyzed the data taken by them. We mention in the discussion that analyzing data from a single entity may be biased. However, the company did not play a role with the study design, analysis, or manuscript preparation. We included all of this information in the new cover letter as well.**
